# Intracerebral hemorrhage and deep microbleeds associated with *cnm*-positive *Streptococcus mutans*; a hospital cohort study

**DOI:** 10.1038/srep20074

**Published:** 2016-02-05

**Authors:** Shuichi Tonomura, Masafumi Ihara, Tomohiro Kawano, Tomotaka Tanaka, Yoshinori Okuno, Satoshi Saito, Robert P. Friedland, Nagato Kuriyama, Ryota Nomura, Yoshiyuki Watanabe, Kazuhiko Nakano, Kazunori Toyoda, Kazuyuki Nagatsuka

**Affiliations:** 1 Division of Neurology, Department of Stroke and Cerebrovascular Diseases, National Cerebral and Cardiovascular Center, 5-7-1 Fujishiro-dai, Suita, Osaka 565-8565, Japan; 2 Departments of Regenerative Medicine and Tissue Engineering, National Cerebral and Cardiovascular Center, 5-7-1 Fujishiro-dai, Suita, Osaka 565-8565, Japan; 3 Division of Cerebrovascular Medicine, Department of Stroke and Cerebrovascular Diseases, National Cerebral and Cardiovascular Center, 5-7-1 Fujishiro-dai, Suita, Osaka 565-8565, Japan; 4 Department of Neurology, University of Louisville, 401 E. Chestnut Street, Suite 510, Louisville, Kentucky, 40292, USA; 5 Department of Preventive Medicine, Kyoto Prefectural University of Medicine, 465 Kajii-cho, Hirokoji Agaru, Kawaramachi-dori, Kamigyo-ku, Kyoto, 602-0841, Japan; 6 Department of Pediatric Dentistry, Osaka University Graduate School of Dentistry, 1-8 Yamada-Oka, Suita, Osaka 565-0871, Japan

## Abstract

Oral infectious diseases are epidemiologically associated with stroke. We previously showed that oral *Streptococcus mutans* with the *cnm* gene encoding a collagen-binding Cnm protein induced intracerebral hemorrhage (ICH) experimentally and was also associated with cerebral microbleeds (CMBs) in our population-based cohort study. We therefore investigated the roles of *cnm*-positive *Streptococcus mutans* in this single hospital-based, observational study that enrolled 100 acute stroke subjects. The *cnm* gene in *Streptococcus mutans* isolated from saliva was screened using PCR techniques and its collagen-binding activities examined. CMBs were evaluated on T2* gradient-recalled echo MRI. One subject withdrew informed consent and 99 subjects (63 males) were analyzed, consisting of 67 subjects with ischemic stroke, 5 with transient ischemic attack, and 27 with ICH. Eleven cases showed *Streptococcus mutans* strains positive for *cnm*. The presence of *cnm*-positive *Streptococcus mutans* was significantly associated with ICH [OR vs. ischemic stroke, 4.5; 95% CI, 1.17–19.1] and increased number of deep CMBs [median (IQR), 3 (2–9) vs. 0 (0–1), p = 0.0002]. In subjects positive for *Streptococcus mutans*, collagen binding activity was positively correlated with the number of deep CMBs (R^2^ = 0.405; p < 0.0001). These results provide further evidence for the key role of oral health in stroke.

Small vessel diseases (SVDs) of the brain (e.g. cerebral microbleeds (CMBs), lacunar infarcts, and white matter lesions) are important biomarkers of vascular injury and burden of brain dysfunction. The underlying mechanisms and risk factors of the SVDs of the brain are poorly understood. CMBs are small round hypo-intense on T2* weighted gradient-recalled echo MRI sequence^1^, histologically corresponding with focal leakage of hemosiderin from abnormal small vessels^2^. Deep CMBs located in the caudate head, lentiform nucleus, internal/external capsules and thalamus are related to hypertensive angiopathy in perforating arterioles, arteriosclerosis and lipohyalinosis^3^. Deep CMBs are strongly associated with the presence of hypertensive intracerebral hemorrhage (ICH) in the same distribution^4^ and are common in recurrent lacunar stroke^5^, supporting the notion that CMBs are markers of cerebral microangiopathy^6^. Risk factors of deep CMBs are long-standing hypertension^5^, advanced age, male sex^7^, and chronic kidney disease^8^; however, deep CMBs may be found in subjects who have no apparent risk factors^9^.

Our recent population-based study showed a strong correlation of CMBs with *cnm* gene positive *Streptococcus mutans* (*cnm*-positive *S. mutans*) in the oral cavity^10^. The adjusted odds ratio for CMBs in the *cnm*-positive *S. mutans* group was 14.4^10^. Consistent with this, Nakano *et al.* found that the *cnm*-positive *S. mutans* contributes to the development of intracerebral hemorrhage by expressing a collagen-binding protein (CBP) that the *cnm* gene encodes on the bacterial surface which disrupts the blood-brain barrier^11^ (BBB). This is in line with the fact that periodontal and other infections have been shown to be risk factors for stroke^12^,^13^. *S. mutans* is a major pathogen of dental caries, and causes bacteremia by dental procedures in daily life^14^. Cell-surface CBP of *S. mutans* mediates its invasion of endothelial cells *in vitro*^15^, and the resultant endothelial failure may underlie CMBs^16^,^17^.

In this hospital cohort study, we aimed to confirm the potential roles of *cnm*-positive *S. mutans* in development of CMBs and ICH in patients with acute cerebrovascular disease, and to explore underlying mechanisms by which this specific pathogen of dental caries directly influences the pathogenesis of SVDs.

## Results

### Background difference between those positive and negative for *cnm*-positive *S. mutans*

One patient withdrew informed consent, and 99 subjects (age 70.1 ± 12.9 years old, 63 male) were subsequently analyzed. *S. mutans* was detected in 51 subjects (52%). Eleven of the 51 subjects (22%) showed positivity for the *cnm* gene. No significant differences were found between those positive for *cnm*-positive *S. mutans* and those negative for *cnm*-positive *S. mutans* in the past medical history of cardiovascular diseases or frequency of vascular risk factors. In terms of results of laboratory blood testing, *cnm*-positive *S. mutans* was significantly associated with higher CRP [0.2 mg/dl (0.1–0.5) vs. 0.1 mg/dl (0–0.2); p = 0.04] and fibrinogen value [361 mg/dl (336–459) vs. 320 mg/dl (274–365); p = 0.01] (Table 1). There were 67 subjects with ischemic stroke (IS), 27 with ICH and 5 with transient ischemic attack (TIA). The 67 subjects with IS consisted of 25 subjects with lacunar stroke (small vessel occlusion), 15 subjects with atherothrombotic stroke (large artery atherosclerosis), 16 subjects with cardiogenic stroke and 11 other known or unknown etiologies. The 27 subjects with ICH consisted of 23 subjects with hypertensive ICH and 4 subjects with probable cerebral amyloid angiopathy (CAA) (Table 1).

### Difference in stroke subtypes between those positive and negative for *cnm*-positive *S.mutans*

**Figure 1:**
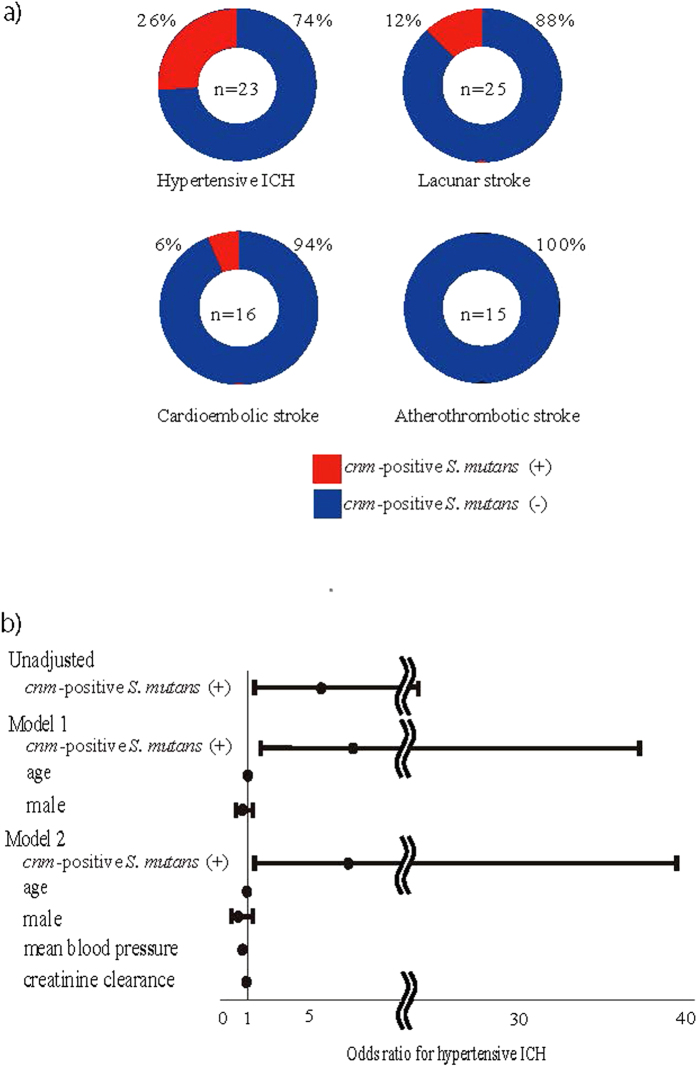
Association of *cnm*-positive *S. mutans* with ICH. (**a**) *cnm*-positive *S. mutans* was detected in 26% of hypertensive intracerebral hemorrhage and 12% of lacunar stroke but in only 6% of cardioembolic stroke and none of atherosclerotic stroke. The odds ratio of *cnm*-positive *S. mutans* in hypertensive ICH versus the other stroke subtypes was 5.56. (**b**) *cnm*-positive *S. mutans* was associated with hypertensive ICH before and after adjusted for established risk factors for ICH, such as age, sex, mean blood pressure and creatinine clearance. Error bars show 95% confidence interval. ICH indicates intracerebral hemorrhage; *S. mutans*, *Streptococcus mutans*.

Six of the 27 subjects with ICH and 4 of the 67 subjects with IS were positive for *cnm*-positive *S. mutans* [odds ratio (OR), ICH vs. IS, 4.5; 95% confidence interval (CI), 1.17–19.1]. The frequency of *cnm*-positive *S. mutans* was greater in those with hypertensive ICH (26%) than those with other stroke types (6%) (Fig. 1a). When analyzed only among subjects with IS, the frequency of *cnm*-positive *S. mutans* was the greatest in those with lacunar stroke (12%) (Fig. 1a). No subjects with probable CAA were found to harbor *cnm*-positive *S. mutan*s. The OR for hypertensive ICH by *cnm*-positive *S. mutans* was 5.56 [95% confidence interval (CI), 1.43–23.9], while it was 7.51 [95% CI, 1.75–37.2] when adjusted for age and sex (model 1) and 7.10 [95%CI, 1.50–39.5] when adjusted for age, sex, mean blood pressure and creatinine clearance (model 2) (Fig. 1b).

### Deep CMBs were significantly greater in subjects with cnm-positive S. mutans

**Figure 2:**
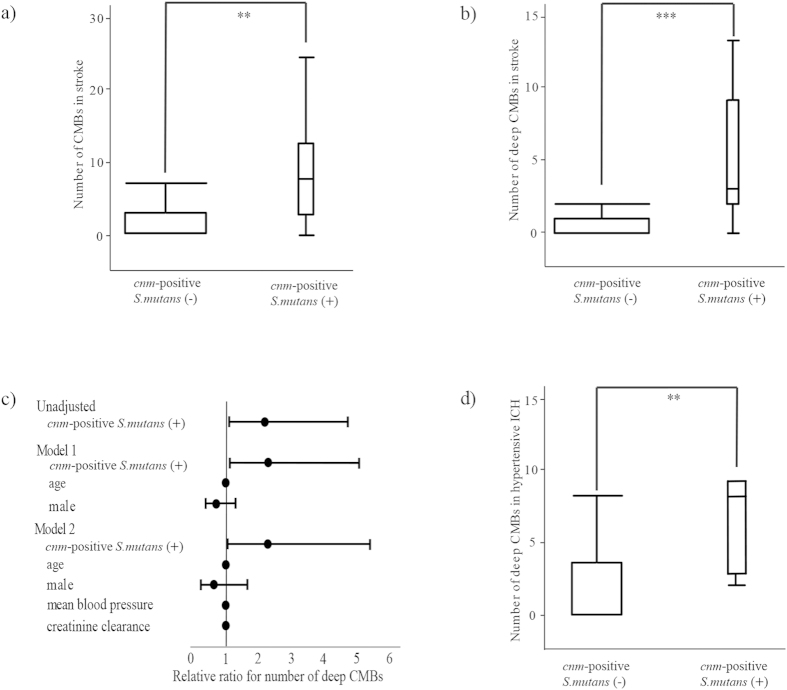
Association of *cnm*-positive *S. mutans* with CMBs. (**a,b**) In total subjects with acute cerebrovascular disease (n = 99), the numbers of CMBs (**a**) and deep CMBs (**b**) were significantly greater in subjects with *cnm*-positive *S. mutans* than those without. (**c**) *cnm*-positive *S. mutans* was associated with deep CMBs before and after adjusted for established risk factors for ICH, such as age, sex, mean blood pressure and creatinine clearance. (**d**) Among subjects with hypertensive ICH (n = 23), the number of deep CMBs was significantly higher in subjects with *cnm*-positive *S. mutans* than those without. Error bars show 95% confidence interval. *S. mutans* indicates *Streptococcus mutans*; CMBs, cerebral microbleeds; ICH, intracerebral hemorrhage. *p < 0.01, **p < 0.001.

We investigated whether *cnm*-positive *S. mutans* is associated with CMBs that are known to be one of the characteristic features of SVDs and often underlie ICH. In 95 subjects whose MRI were available (4 subjects contraindicated due to their pacemakers), CMBs were found in 53 (56%) which comprised of 9 of the 11 subjects with *cnm*-positive *S. mutans* (81%) and 44 of the other 84 subjects (52%), with a marginal intergroup difference (p = 0.06). Total number of CMBs was significantly higher in subjects with *cnm*-positive *S. mutans* compared to those without (median (IQR), 8 (3–13) vs. 0.5 (0–4); p = 0.002) (Fig. 2a). The relative ratio for total CMBs with *cnm*-positive *S. mutans* compared to those without was 1.93 [95% CI, 1.06–3.88]. In terms of their locations, the number of deep CMBs was significantly higher in subjects with *cnm*-positive *S. mutans* compared to those without (3 (2–9) vs. 0 (0–1); p = 0.0002) (Fig. 2b). The relative ratio for number of deep CMBs with *cnm*-positive *S. mutans* compared to those without was 2.2 [95% CI, 1.1–4.7], while it was 2.3 [95% CI, 1.1–5.1] when adjusted by the model 1 and 2.3 [95% CI, 1.1–5.3] when adjusted by the model 2 (Fig. 2c). Such a significant difference was also seen among the ICH subjects depending on the presence or absence of *cnm*-positive *S. mutans* (8 (2.8–9.0) vs. 2 (0–8); p = 0.0037) (Fig. 2d). In contrast, the score of the lobar and infratentorial CMBs were not significantly different between the two groups (lobar CMBs, 0 (0–4) vs. 1 (0–1); p = 0.43; infratentorial CMBs, 0 (0–3) vs. 0 (0–0.8); p = 0.60). When the number of total or deep CMBs was categorized into 0 (none), 1 or 2 (few), and ≥ 3 (multiple) groups, there were significant differences in the positive rate of *cnm*–positive *S. mutans* among the three groups (p < 0.01 for total CMBs; p < 0.001 for deep CMBs): the group with multiple CMBs showed significantly greater rate of *cnm*–positive *S. mutans* (Table 1).

### Collagen binding activity of *cnm*-positive *S. mutans* are correlates with number of deep cerebral microbleeds

**Figure 3:**
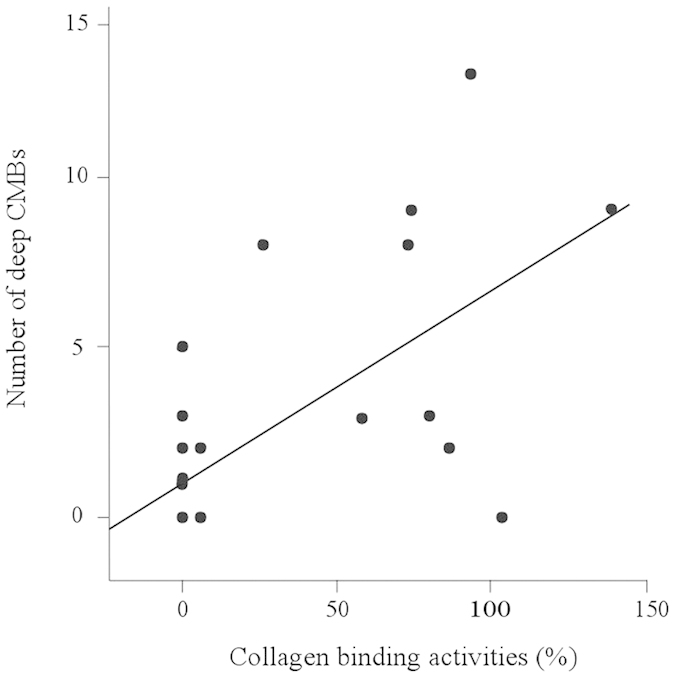
Correlation between collagen binding activity of *S. mutans* and number of deep CMBs. The collagen-binding activity of *S. mutans* significantly correlated with the number of deep CMBs (n = 50, γ = 0.054, P < 0.0001, R2 = 0.405) after one extreme outlier was excluded by using jackknife technique. *S. mutans*, *Streptococcus mutans*; CMBs, cerebral microbleeds.

The collagen-binding activity was measured for the 12 strains of *S. mutans* from 11 subjects who were positive for *cnm*. The collagen-binding activity of *cnm*-negative *S. mutans* retrieved from subjects with deep CMBs was also measured, which were not detectable in most of cases, as reported in other studies. Collagen binding activity was correlated with numbers of deep CMBs (γ = 0.054; R2 = 0.405; p < 0.0001) (Fig. 3) after one extreme outlier was identified by jackknife test and excluded from the analysis because the number of deep CMBs was more than median plus 5SD.

### Short case report of recurrent ICH with *cnm*-positive *S. mutans*

**Figure 4:**
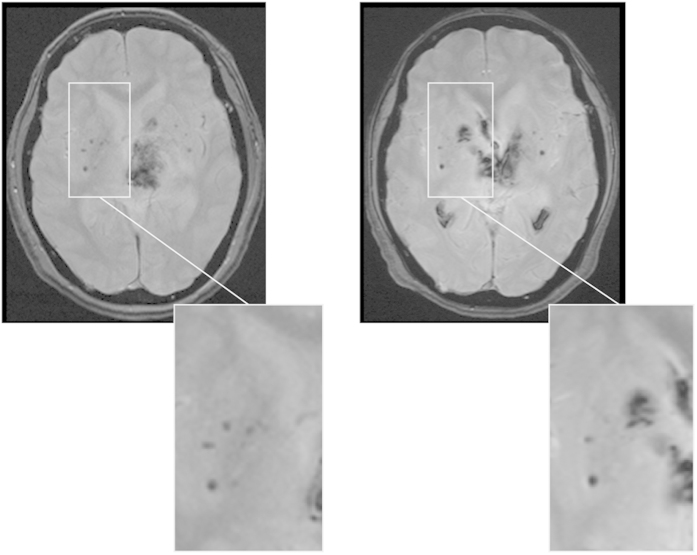
Recurrent ICH occurred in a patient with *cnm*-positive *S. mutans.* A 57-year-old woman with left putaminal hemorrhage (left) had been found to have *cnm-*positive *S. mutans* and had a recurrent hemorrhage in the right caudate nucleus with only one-year interval (right) despite good hypertension control.

A 57-year-old woman with left putaminal hemorrhage was found to have *cnm*-positive *S. mutans* and had a recurrent hemorrhage in the right putamen within a one-year interval despite good control of hypertension with medications and absence of other apparent risk factors, such as hyperlipidemia, diabetes mellitus, smoking or alcohol abuse. The location of the recurrent hemorrhage corresponded to the location of CMBs that had been detected in the previous T2* gradient-recalled echo (GRE) MRI on the first admission, and the number of deep CMBs increased on the second admission (Fig. 4). The isolation rate of *cnm*-positive *S. mutans* strains in the five cultured plates was increased (first vs. second admission; 1/5 vs. 5/5). The collagen binging activities of *cnm*-positive *S. mutans* were 76% and 111% in the first and second isolation, respectively. These findings suggest a poor prognosis, or a higher tendency for recurrent bleeding in subjects with *cnm*-positive *S. mutans* strains.

## Discussion

This study conducted in our acute stroke cohort showed a significant correlation of *cnm*-positive *S. mutans* with hypertensive ICH and deep CMBs, two of the major hemorrhagic phenotypes of arteriolosclerosis in the perforating arteries. Other forms of SVDs, including white matter hyperintensity, lobar CMBs and lacunar infarctions, did not show associations with *cnm*-positive *S. mutans*. In addition, the relatively increased positivity of *cnm*-positive *S. mutans* in subjects with lacunar infarction (small vessel occlusion) compared to other IS subtypes (Fig. 1) further suggests the linkage between *cnm*-positivity and small vessel changes. The elevated levels of two inflammatory markers, CRP and fibrinogen, in subjects with *cnm*-positive *S. mutans* suggest that inflammation can be derived from the *cnm*-positive *S. mutans* infection, as the bacteria latently found in the dental caries can cause daily bacteremia^14^. The correlation of the collagen-binding activity with the number of deep CMBs suggests a pathogenic role for CBP expressed on the cell surface of *S. mutans* strains in the development of hemorrhagic phenotype of SVD. Although both inflammation and endothelial damage are thought to contribute to small vessel disease^16^, little is known about specific mechanism underlying CMBs^17^. Therefore, infection with *cnm*-positive *S. mutans* may explain the causation of inflammation and endothelial damage as underlying etiologies of CMBs.

Our study shows a possible mechanism by which *cnm*-positive *S. mutans* induces hemorrhagic changes in perforating arterioles. The permeability of the BBB is known to increase with age^18^ as a result of loss of endothelial integrity. Long-standing hypertension further affects the structure of small vessels leading not only to BBB disruption but also to accumulation of type I collagen^19^. Dental caries results in the destruction of enamel on the tooth surface enabling *cnm*-positive *S.mutans* access to bloodstream^20^,^21^. Age and hypertension related vascular changes in cerebral small vessel disease may release vascular factors that attract concentration dependent collagen binding factors^22^, including those of *cnm*-positive *S. mutans*. Once *cnm*-positive *S. mutans* attach to the exposed collagen of perforating arterioles, infiltration of neutrophils may aggravate local inflammation, resulting in increasing permeability of the BBB^23^ and higher delivery of enzymes, such as myeloperoxidase^16^, to accelerate endothelial damage. Furthermore, the negative charges of the bacterial surface proteoglycans may prevent negatively charged platelets from interacting with collagen^11^,^24^, which will impair primary hemostasis resulting in prolongation of focal hemorrhage. There is an emerging concept of the brain-gut axis. The brain-gut axis and its association with several disorders including cardiovascular disease^25^ and neurodegenerative disorders^26^ has been considered mainly in regard to the flora in the stomach and lower intestine. Since *S. mutans* is a major pathogen of oral caries, our data provides a new concept of brain-oral association. Future experimental work should focus on determining how oral infectious pathogens induce or aggravate CMBs and CAA. Furthermore, *S. mutans* with CBP has some mechanistic implications in other systemic disorders including infective endocarditis^15^, ulcerative colitis^27^ and IgA nephropathy^28^. The strong correlation between highly virulent *S. mutans* and systemic disease including CMBs/ICH may provide a new breakthrough for future prevention and intervention.

Some limitations should be addressed. First, the isolation rate of *S. mutans* strains was lower than that from normal volunteers. This may be associated with better oral hygiene after the oral care the stroke subjects received in the acute phase or with oral drying because of the comorbidities of stroke prior to sampling of oral plaque in this study. Alternatively, the lesser number of teeth in the aged stroke subjects may have affected the lower isolation rate of *S. mutans* strains because *S. mutans* only resides in hard tissue, such as tooth. It should be determined whether stroke subjects have lower isolation rates of *S. mutans* with thorough screening tests in consideration of future prevention strategy of cerebral hemorrhagic disorders. Second, MRI images were taken with 1.5-tesla and 3-tesla MR scanners interchangeably because of the issue of day and night availability of MR scanners in the acute settings. However, the ratio of usage of 3-tesla MRI images was not different between *cnm*-positive and -negative subjects, minimizing the possibility that the resolution of MRI images affected the results. The third limitation was that this cross-sectional study did not determine the exact interval between infection of *cnm*-positive *S. mutans* and appearance of CMBs. Finally, this study enrolled a relatively small number of stroke patients from single ethnic group. This study should therefore be viewed as hypothesis generating although the data certainly merits further study to investigate the difference in frequency of *cnm*-positive *S. mutans* and the impact of the oral bacteria on pathogenesis of stroke in different ethnic groups.

In summary, Cnm proteins of *S. mutans* may be associated with development of deep CMBs and ICH with a mechanistic link to chronic inflammation.

## Materials and Methods

### Participants

One hundred subjects admitted to the National Cerebral and Cardiovascular Center (NCVC) because of acute IS, TIA and ICH between February and August 2014 were enrolled. All subjects underwent neurological examination by National Institute of Health Stroke Scale (NIHSS) score and modified Rankin scale pre-admission, at discharge and 3 months after discharge. Those with disturbances of consciousness were not enrolled because of difficulty of oral sampling. IS was classified using the trial of Org 10172 in Acute Stroke Treatment (TOAST) classification^29^, ICH was classified by the structural vascular lesion; medication; CAA; systemic disease; hypertension; undetermined (SMASH-UICH) classification^30^ and TIA was defined as related syndrome of stroke symptoms that resolve completely within 24 hours. The diagnosis of probable CAA was established by the modified Boston criteria^31^. As traditional vascular risk factors, we recorded hypertension, diabetes, dyslipidemia, atrial fibrillation (all medically diagnosed and/or on relevant drugs), ever smoker and drinking (interview on admission). We collected past medical history of IS, ICH, and ischemic heart disease in clinical records. Laboratory tests included complete blood counts, metabolite profile and blood coagulation.

### Sample collection

Oral saliva and dental plaque specimens were collected from the subjects in the first 3 days following admission.

### Culture condition of *Streptococcus mutans*

Oral samples were inoculated on Mitis-Salivarius medium with bacitracin (MSB, 100 U/ml; Sigma-Aldrich, St. Louis, MO, USA) and 15% sucrose (MSB agar) and anaerobically incubated at 37 centigrade for 48 hours. *Streptococcus mutans* strains were isolated morphologically and all strains were anaerobically grown in brain heart infusion (BHI) broth (Difco Laboratories, Detroit, MI, USA) at 37 centigrade for 24 hours.

### *cnm* gene positivity and collagen binding assay

After cultured in BHI broth, DNA of each strain was extracted. *S.mutans* and *cnm* gene encoding CBP was screened using polymerase chain reaction techniques. MKD primer^32^ was used to detect *S.mutans* and *cnm* primer was used to identify *cmm* gene^33^. A collagen-binding assay with type I collagen was conducted to examine collagen-binding activities of each isolated *S. mutans* strain according to the method described by Waterhouse and Russell^34^, with some modifications^32^. The activities was evaluated under fixed conditions of 1 mg of type I collagen and 1 ×10^10^ bacterial cells. The activities for each strain are expressed as a percentage compared with the positive control *S. mutans* TW871 which has known binding activity to type I collagen^32^ as 100%. These experiments were conducted by a researcher (R.N.) who was blinded to clinical information.

### Imaging acquisition

Magnetic resonance imaging (MRI) was performed either with 1.5 Tesla (Magnetom Sonata or Vision; Siemens Medical Solutions, Erlangen, Germany) or 3.0 Tesla (Magnetom Verio or Spectra; Siemens Medical Solutions, Erlangen, Germany) scanners. A standardized protocol was employed for all time points that included DWI and apparent diffusion coefficient (ADC), fluid attenuated inversion recovery (FLAIR) and T2* GRE.

### Imaging analysis

The number and location of CMBs and severity of white matter lesions were analyzed. Cerebral microbleeds were evaluated on T2* GRE MRI and were defined according to the published criteria^1^. The locations of CMBs were categorized into lobar (cortical gray or subcortical white matter), deep (deep gray matter in the basal ganglia and thalamus; or white matter in the corpus callosum, internal, external, and extreme capsule) and infratentorial (cerebellum and brainstem) regions. The number and location of CMBs were rated by one neurologist (S.T.) and independently by another neurologist (Y.O.). Inter-rater correlation coefficients were 0.97 for deep, 0.97 for lobar and 0.90 for infratentorial CMBs.

### Ethics regarding human subjects

All subjects or their family members provided written informed consent and the study was approved by the Institutional Ethical Committee of the National Cerebral and Cardiovascular Center. The methods were carried out in accordance with the Ethical Guidelines for Medical and Health Research Involving Human Subjects.

### Statistical analyses

For statistical analyses, JMP 11 software (SAS Institute Inc., Cary, NC) was used. Differences in dichotomous variables were analyzed using Chi-square test. Wilcoxon signed rank test was used to analyze differences in the median of continuous variables between groups. Multivariable models were generated to determine the contribution of *cnm*-positive *S. mutans* to hypertensive ICH and number of deep CMBs after adjusting for known risk factors (age, sex, mean blood pressure, and creatinine clearance). To further determine the association between *cnm*-positive *S. mutans* and CMBs, patients were categorized into three groups according to the number of total or deep CMBs; 0 (none), 1 or 2 (few), and ≥3 (multiple) groups, and intergroup differences were assessed with Pearson’s chi-square test. To assess the relationship between collagen binding activity of *S. mutans* and deep CMBs, correlation analysis was used after the jackknife test was applied to exclude outliers. Statistical significance level was set at 0.05 for all analyses. Subjects without *cnm*-negative *S. mutans* included both those positive for *cnm*-negative *S. mutans* and those negative for *S. mutans*.

## Additional Information

**How to cite this article**: Tonomura, S. *et al.* Intracerebral hemorrhage and deep microbleeds associated with *cnm*-positive *Streptococcus mutans*; a hospital cohort study. *Sci. Rep.* **6**, 20074; doi: 10.1038/srep20074 (2016).

## Figures and Tables

**Table 1:** Baseline characteristics of patients.

Characteristics		Total, n = 99		*cnm*-positive *S. mutans* (+), n = 11		*cnm*-positive *S. mutans* (−), n = 88		p-value
Age		70.1 ± 12.9		74.5 ± 10.1		69.5 ± 10.1		0.22
Gender, M:F		63:36		6:5		57:31		0.51
**Cardiovascular risk factors, No. (%)**								
Hypertension		82	(82.8)	10	(90.9)	72	81.2	0.45
sBP (mmHg)		174	(158–195)	174	(163–203)	174	(157–193)	0.28
dBP (mmHg)		94	(87–107)	103	(83–109)	93	(87–107)	0.61
Diabetes mellitus		17	(17.1)	3	(27.3)	14	(15.9)	0.35
Hyperlipidemia		34	(34.3)	6	(54.5)	28	(31.8)	0.13
Ever Smoker		45	(45.5)	3	(27.3)	42	(47.7)	0.19
**Past medical history, No. (%)**								
Ischemic stroke		21	(21.2)	3	(27.3)	24	(27.3)	0.60
Intracerebral hemorrhage		8	(8.1)	0	(0.0)	8	(9.1)	0.30
**Laboratory examination**								
CCr, ml/min		55	(43–73)	50	(38–78)	56	(43–73)	0.49
CRP, mg/dl		0.1	(0.0–0.2)	0.2	(0.1–0.5)	0.1	(0.0–0.2)	0.04
WBC, (×1000)/μl		6.4	(5.2–8.1)	6.5	(4.6–7.4)	6.3	(5.2–8.2)	0.92
Fibrinogen, mg/dl		326	(275–371)	361	(336–459)	320	(274–365)	0.01
**Classification of stroke, No. (%)**								
Ischemic Stroke		67	(67.7)	4	(36.4)	63	(71.6)	0.02
Large artery atherosclerosis		15	(15.2)	0	(0.0)	15	(17.0)	0.14
Cardioembolism		16	(16.2)	1	(9.1)	15	(17.0)	0.50
Small vessel occlusion		25	(25.3)	3	(27.3)	22	(25.0)	0.87
Other determined etiology		2	(2.0)	0	(0.0)	2	(2.2)	0.61
Unknown etiology		9	(10.0)	0	(0.0)	9	(10.2)	0.27
Intracerebral hemorrhage		27	(27.3)	6	(54.5)	21	(23.9)	0.03
Hypertensive ICH		23	(23.3)	6	(54.5)	17	(19.3)	<0.01
CAA		4	(4.0)	0	(0.0)	4	(4.5)	0.47
TIA		5	(5.1)	1	(9.1)	4	(4.5)	0.42
**SVDs markers, No. (%)**								
Total CMBs	0	46	(46.5)	2	(18.2)	44	(50.0)	<0.01
	1 or 2	18	(18.2)	0	(0.0)	18	(20.5)	
	≥3	356	(35.4)	9	(81.8)	26	(29.5)	
Deep CMBs	0	59	(59.6)	2	(18.2)	57	(64.8)	<0.001
	1 or 2	19	(19.2)	2	(0.0)	17	(19.3)	
	≥3	21	(21.2)	7	(81.8)	14	(15.9)	
DSWMH>grade 2		13	(13.7)	2	(18.2)	11	(13.1)	0.64
PVWMH>grade 2		20	(21.5)	2	(18.2)	18	(21.4)	0.80

Chi-square test or Wilcoxon test were conducted. Age is expressed as mean (±standard deviation) and the other variables are expressed as median (interquartile range). F, female; M, male; CAA, cerebral amyloid angiopathy; CCr, creatinine clearance; CMBs, cerebral microbleeds; CRP, c-reactive protein; dBP, diastolic blood pressure; DSWMH, deep subcortical white matter hyperintensity; ICH, intracerebral hemorrhage; PLT, platelet; PVWMH, periventricular white matter hyperintensity; sBP, systolic blood pressure; SVDs, small vessel diseases; TIA, transient ischemic attack; WBC, white blood cell.
